# Identifying genetic targets in clinical subtypes of Parkinson’s disease for optimizing pharmacological treatment strategies

**DOI:** 10.1038/s41392-024-02020-x

**Published:** 2024-11-18

**Authors:** Dewen Kong, Cao Li, LingYan Ma, Lida Du, Nan Jiang, Xiaoyue Zhao, Sen Zhang, Zhigang Zhao, Lianhua Fang, Guanhua Du

**Affiliations:** 1 State Key Laboratory of Bioactive Substances and Functions of Natural Medicines, Beijing Key Laboratory of Drug Target Identification and Drug Screening, Institute of Materia Medica, Chinese Academy of Medical Sciences and Peking Union Medical College, Beijing, China; 2 Department of Pharmacy, Beijing Tiantan Hospital, Capital Medical University, Beijing, China; 3 Center for Movement Disorders, Department of Neurology, Beijing Tiantan Hospital, Capital Medical University, Beijing, China; 4 China National Clinical Research Center for Neurological Diseases, Beijing, China; 5 Institute of Molecular Medicine & Innovative Pharmaceutics, Qingdao University, Qingdao, China; 6 School of Pharmacy, Henan University, Kaifeng, China; 7 Medical Science Research Center, Peking Union Medical College Hospital, Chinese Academy of Medical Science and Peking Union Medical College, Beijing, China

**Keywords:** Molecular neuroscience, Neurogenesis

## Abstract

The heterogeneity of Parkinson’s disease (PD) has been recognized in clinical, with patients categorized into distinct subsets based on motor phenotype, such as tremor-dominant PD (TD), postural instability and gait difficulty-dominant PD (PIGD) and mixed PD (Mix). Despite this categorization, the underlying mechanisms of this heterogeneity remain poorly understood, and there is no personalized effective treatment for each PD subtype. To address this, a rat model for PD subtypes was established by unilateral stereotaxic injection of 6-OHDA, followed by cluster analysis of behavioral data. The serum neurofilament light chain (NfL) and uric acid (UA) levels as well as alterations in brain autonomic activity in rats were consistent with clinical patients, and metabolomics results showed that more than 70% of the metabolites in the serum of different subtypes of PD rats and clinical patients appeared to be consistently altered. Further transcriptomic analysis by RNA-seq has elucidated that the development of PD subtypes is associated with altered gene expression in neurotransmitter, neuronal damage in the central or peripheral nervous system, and lipid metabolism. In addition, based on the subtype-specific differentially expressed genes, 25 potential drug candidates were identified. Notably, the Alox15 inhibitor baicalein showed a greater efficacy on Mix rats, highlighting the possibility of selecting targeted treatments for well-defined individuals.

## Introduction

Parkinson’s disease (PD) is one of the most prevalent age-related neurodegenerative disorders, affecting ~1% of individuals over the age of 60 and 0.3% of the general population.^1,2^ The diagnosis of PD is commonly based on motor symptoms, which include tremor, akinesia, and rigidity. Increasing evidence suggests that PD is a heterogeneous neurodegenerative disorder with variable clinicopathologic phenotypes, rather than a single disease entity. Accumulating reports have classified PD into subtypes based on its clinical features, as delineated in part II and III of the Unified Parkinson’s Disease Rating Scale as the postural instability and gait difficulty-dominant PD (PIGD), tremor-dominant PD (TD) or mixed PD (Mix) subtypes.^3,4^ Current research indicates that the TD subtype is a favorable prognostic indicator, with patients generally experiencing a higher quality of life than those with other subtypes.^5–8^ Correspondingly, post mortem analysis of TD patients have revealed significantly reduced cortical lewy body deposition in the brain,^9^ as well as lower concentrations of acetylcholine (Ach) and neurofilament light chain (NfL) in the serum.^10^ Besides, previous functional neuroimaging studies have demonstrated that structural brain damage and volume reduction are more significant in patients with the PIGD subtypes. From a neurocircuitry perspective, dysfunction of the striatal-thalamo-cortical (STC) loop accounts for akinesia and rigidity,^11^ while resting tremor is associated with altered activity in both the STC pathway and the cerebello-thalamo-cortical (CTC) circuit.^12,13^

The symptomatic heterogeneity in different PD subtypes has distinct pathophysiological bases. Uncovering these mechanisms is critical for enhancing our understanding of PD and development of effective therapies.^14^ Subtyping PD is beneficial for facilitating PD research, management, and patient counseling regarding prognosis. The science of PD subtype is currently developing in more physiologically based directions, which has also been proposed as one of the four major issues in the development of PD.^15^ Clinical testing of interventions aimed at identifying disease-modifying medications has so far shown mostly poor results, perhaps due to differences in the pathophysiological mechanisms contributing to PD among patients. Thus, an understanding of the subtype hopefully brings us steadily getting closer to achieving truly disease-modifying therapies for PD. However, only a small number of research offers supportive evidence on serum biomarkers and neuroimaging, the possible pathophysiologic mechanisms responsible for clinical and neuropathologic heterogeneity are still unclear.

Considering the broad spectrum of symptoms and prognosis, as well as the variability responses and tolerability of patients to current symptomatic treatments, the future direction of PD research might be the development of subtype-specific therapies to advance personalized medical treatment of PD.^16^ Previous analyses have predominantly based on clinical features, such as serum/cerebrospinal fluid biomarkers and neuroimaging, and have been limited by insufficient longitudinal assessments to explore the mechanisms underlying PD subtypes.^5,17^ Furthermore, the pharmacogenomics is a fast growing science that links genetic variations to specific drugs response, it has not yet been used in routine clinical practice in PD subtypes. Animals have been widely used in preclinical basic research and drug screening due to their irreplaceable advantages. Currently, there is no published attempt to classify subtypes of PD in animals. Our research indicates that rats unilateral injection of 6-OHDA exhibit a clinically comparable heterogeneity in motor behavior, which may be related to the location and degree of lesion, or the tolerance and habituation of the animals. Furthermore, we are particularly interested in elucidating the molecular basis and drug development regarding the subtypes of Parkinson’s Disease.

To identify potential candidates for the precise treatment of PD subtypes, we conducted cluster analysis on a comprehensive baseline dataset. This dataset included results from the rotarod test, open field test, neurological function test and tremor monitoring to establish rat models for TD, PIGD and Mix subtypes. In addition, we compared the changes of serum biomarkers, metabolites and neural activity in different subtypes of PD rats and clinical patients to clarify the consistency between the rat model and clinical subtypes. By performing transcriptomic analysis of rat substantia nigra, we further investigated the genetic variants among different subtypes of PD including neurotransmitter function, neuronal damage in both central and peripheral nervous systems, lipid metabolism and prognosis. To the best of our knowledge, our methodology presents a novel and easily generalizable approach for robust subtype identification by incorporating behavioral scores of PD rats symbolizing different clinical symptoms, based on which therapeutic targets and potential therapeutic candidates for different subtypes of PD are obtained. This provides an opportunity for subsequent refined preclinical studies of the etiology of PD subtypes and studies of the treatment responsiveness of existing anti-PD drugs to different subtypes. Additionally, we demonstrate that our candidate drug, baicalein, offers superior protective effect on Mix subtype rats. This study aims to bridge the gap between the molecular mechanisms and drug development of PD and the clinical subtypes.

## Results

### Establishment of the motor subtype PD rat model

**Fig. 1:**
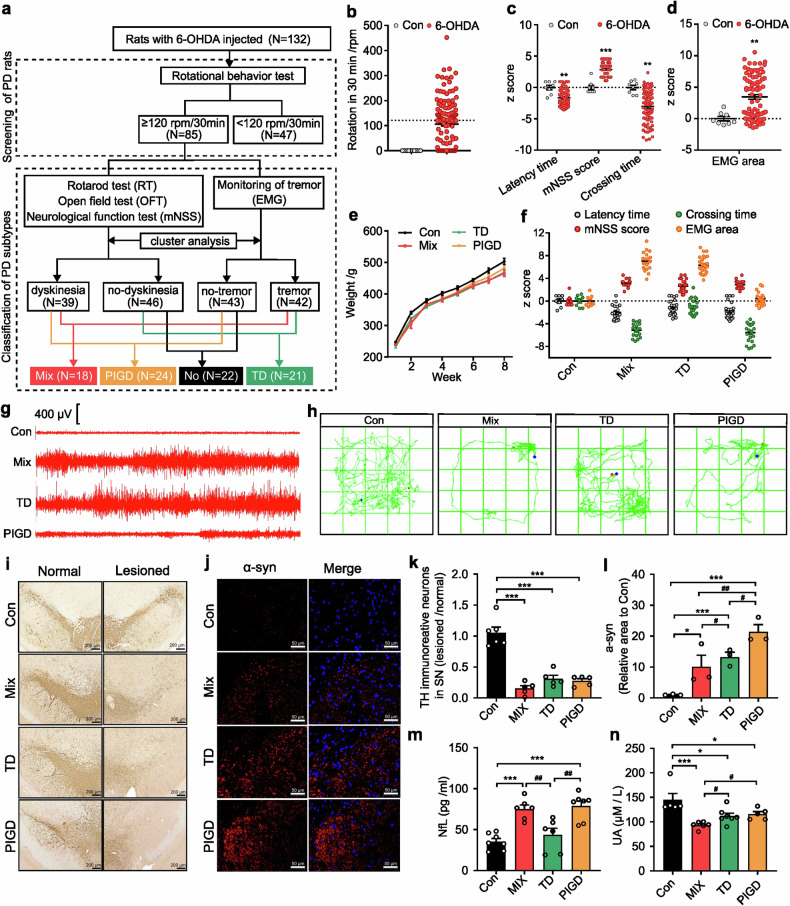
Establishment of the rat model for different PD subtypes. **a** The schematic diagram of the experimental protocols. **b** Rotational behavior test. **c** Athletic ability evaluation including rotarod test, neurological function test and open field test. **d** electromyography (EMG) for tremor monitor. **e** Body weight. **f** Behavioral data of different subtypes of PD rats for classification. **g** Segments of tremor monitor activity profiles. **h** The movement routes. **i**, **k** Representative images and quantitative analysis of TH immunohistochemical staining in normal and lesioned SN of rats. **j**, **l** Representative images and quantitative analysis of α-syn immunofluorescence staining in lesioned SN of rats. **m**, **n** NfL and UA concentration in serum. The data described are Mean ± SEM, n = 5–132. *p < 0.05, **p < 0.01 and ***p < 0.001 vs. control; ^#^p < 0.05, ^##^p < 0.01 and ^###^p < 0.001 vs. other subtype

This study involved 10 control rats and 132 6-OHDA-lesioned rats (Fig. 1a). Rats that achieved at least four rotations per minute were considered PD rats for subsequent behavioral tests (Fig. 1b). PD rats showed heterogeneity in motor symptoms, including latency time, mNSS score, crossing times (Fig. 1c) and tremor (Fig. 1d). According to Fig. 1a, composite z-scores of the rotarod test, open field test and neurological function test were clustered using SPSS 22.0 software. PD rats were classified as either dyskinesia or no-dyskinesia groups. Similarly, z-scores of the EMG area associated with tremor were clustered to classify PD rats as tremor or no-tremor. Based on this, PD rats with both dyskinesia and tremor symptoms were categorized as mixed PD (Mix), while those exhibiting only dyskinesia or tremor were classified as postural instability and gait difficulty-dominant PD (PIGD) or tremor-dominant PD (TD) respectively (Fig. 1f–h). In addition, we found there was no significant difference in body weight between control rats and each subtype of PD rats (Fig. 1e). As shown in Fig. 1i, k, TH-positive cells decreased in all three subtypes of PD rats, but no significant differences were observed. Conversely, alpha-synuclein (α-syn) was most highly expressed in the substantia nigra of PIGD rats (Fig. 1j, l).

### Evaluation of the consistency of motor subtype PD rat model with the clinical observations

**Fig. 2:**
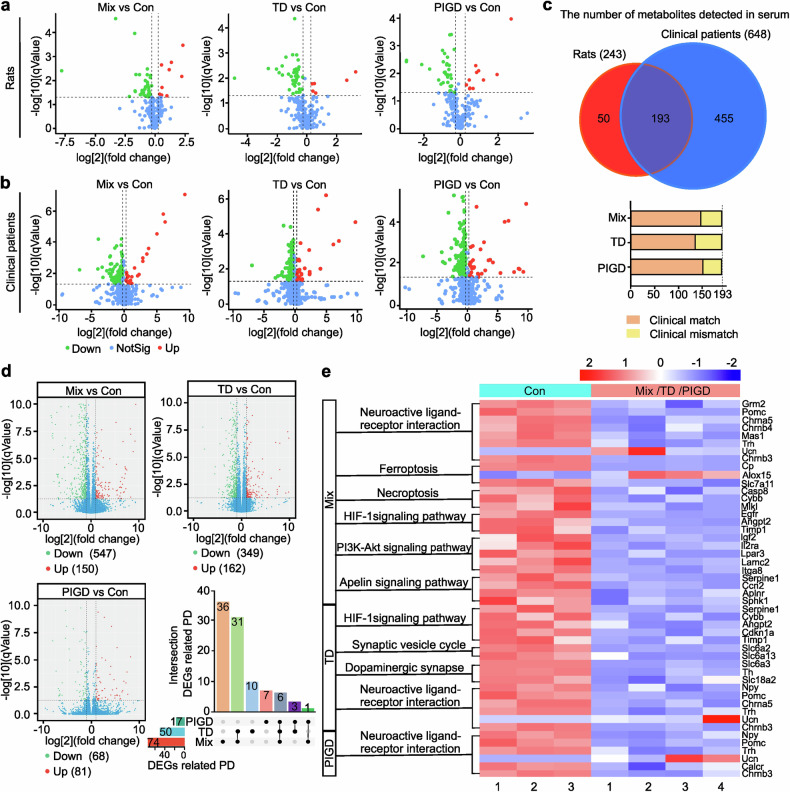
Metabolomic analysis and transcriptomic analysis of rats and patients with different subtypes of PD. **a**, **b** Volcano plots of metabolites detected in the serum of rats and clinical patients. **c** The number of metabolites with consistent or inconsistent changes in rats and clinical patients serum. **d** Differences of gene expression in Volcano plot and the intersection of DEGs related to PD. **e** KEGG pathways about neurological disorders. n = 3–6

Elevated plasma neurofilament light chain (NfL) and uric acid (UA) were associated with accelerated motor progression.^12,18,19^ Our findings indicated a statistically significant increase in NfL concentration in the serum of Mix and PIGD rats (Fig. 1m). Correspondingly, a reduction in serum UA levels was observed in PD rats, with the lowest concentrations detected in the Mix subtype, whereas no significant differences were found between the TD and PIGD subtypes (Fig. 1n). We utilized resting-state functional magnetic resonance imaging (rs-fMRI) combined with the amplitude of low-frequency fluctuation (ALFF) method to investigate the modulations of neural activity across different PD rat subtypes. Previous findings underscore the differential involvement of the striatal-thalamo-cortical (STC) and cerebello-thalamo-cortical (CTC) circuits underlying the TD and PIGD subtypes of PD, but the existing studies have not reached a unified conclusion.^20,21^ Here, main areas with decreased ALFF were also detected in TD and Mix rats, including striatum, thalamus, cortex and poaterior lobe of cerebellum. Additionally, we found that TD and Mix rats showed decreased ALFF mainly in right cortical areas, while TD and PIGD subtypes of PD rats exhibited lower ALFF in the left sub-cortical areas (medulla oblongata, thalamus, striatum, cerebellum, cingulate gyrus), which may be related to the unilateral damage caused by 6-OHDA (Supplementary Fig. 1, Supplementary Table 3). However, no significant laterality was detected in sub-cortical brain regions with reduced ALFF in Mix rats, which is involved in the STC and STC loops. Furthermore, metabolomic analyses were conducted on serum samples from both rats and clinical patients, resulting in the detection of 243 and 648 metabolites, respectively, with 193 metabolites that were detected in both rat and human (Fig. 2a–c). Of these, 76.68% (Mix vs control), 78.76% (TD vs control), 70.47% (PIGD vs control) metabolite alterations in rat serum matched with that in clinical patients. These findings support the consistency between rats and clinical patients with different subtypes of PD.

### Analysis of DEGs and KEGG enrichment in different subtypes of PD rats

To further explore the underlying mechanisms contributing to the heterogeneity of PD, namely the mechanism of subtypes, 15 cDNA libraries (3 control samples, 4 Mix, 4 TD and 4 PIGD subtype of PD, respectively) were established and sequenced. Different expressed genes (DEGs) between the control group and each PD subtype group were identified from the RNA sequencing data. Raw sequence data reported in this paper have been deposited (PRJCA031168) in the Genome Sequence Archive in the BIG Data Center, Chinese Academy of Sciences under accession codes CRA019617 for RNA sequencing data that are publicly accessible at http://bigd.big.ac.cn/gsa. The results showed that 547 (Mix vs control), 349 (TD vs control), 68 (PIGD vs control) down-regulated genes, and 150 (Mix vs control), 162 (TD vs control), 81 (PIGD vs control) up-regulated genes (Fig. 2d). Notably, only 10.62%, 9.78%, and 11.41% of DEGs in the Mix, TD and PIGD subtypes were recognized as PD-associated genes by gene-disease association (GDA) analysis, with 36 DEGs especially associated with Mix, 10 with TD, and 7 with PIGD (Fig. 2d). As shown in Fig. 2e, PD-associated DEGs were further analyzed for Kyoto Encyclopedia of Genes and Genomes (KEGG) pathway enrichment, and pathways related to neurological disorders being highlighted. KEGG enrichment results indicated that all PD subtypes were involved in neuroactive ligand-receptor interaction, while the Mix and TD subtypes were additionally related to HIF-1 signaling pathway. In addition, Mix subtype was specifically correlated with ferroptosis, necroptosis, PI3K-AKT and apelin signaling pathway, while TD subtype was associated with the synaptic vesicle cycle and dopaminergic synapses.

### Subtypes of PD differ in neuronal development and differentiation

**Fig. 3:**
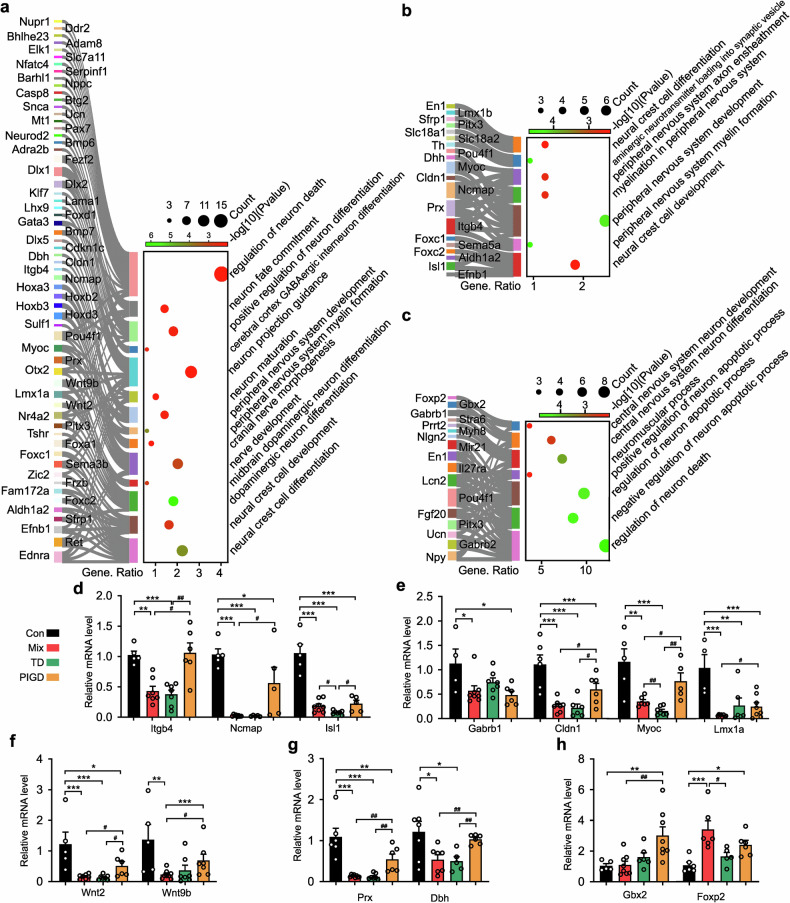
Subtypes of PD differ for neuronal development and differentiation. The biological function of neuronal development and differentiation in Mix (**a**), TD (**b**) and PIGD (**c**) subtypes of PD rats. **d**–**h** Relative mRNA levels of Itgb4, Drx, Ncmap, Cldn1, Myoc, Dbh, Foxp2, Gbx2, Isl1, Gabrb1, Wnt2, Lmx1a and Wnt9b detected by RT-PCR. Data described are Mean ± SEM, n = 3-6. *p < 0.05, **p < 0.01 and ***p < 0.001 vs. control. ^#^p < 0.05, ^##^p < 0.01 and ^###^p < 0.001 vs. other subtype

Consistent with previous reports,^22^ the biological processes (BP) of GO enrichment indicated disruptions in neuronal development, differentiation and death in different subtypes of PD rats were all perturbed. Importantly, our findings revealed for the first time that the TD subtype was mainly associated with peripheral nervous system (PNS) damage, encompassing processes such as PNS myelin formation, PNS development, myelination in the PNS and PNS axon ensheathment (Fig. 3b). Conversely, the symptoms associated with the PIGD subtype may be attributable to central nervous system (CNS) disorders, including disruptions in CNS neuron differentiation and development (Fig. 3c). As expected, Mix subtype PD rats, characterized by both tremor and dyskinesia, demonstrated pathological alterations in both central and peripheral neurons. These alterations encompass mid-brain dopaminergic neuron differentiation, cranial nerve morphogenesis, cerebral cortex GABAergic interneuron differentiation, peripheral nervous system, and overall peripheral nervous system development (Fig. 3a).

The Real-Time PCR (RT-PCR) validation results demonstrated genes related to PNS were down-regulated in Mix and TD subtypes of PD rats, including Itgb4, Prx, Ncmap, Cldn1, Myoc, and Dbh (Fig. 3d, e, f). Conversely, Foxp2 (Fig. 3h), linked to the CNS, was significantly up-regulation in the Mix and PIGD subtypes. Although these findings were not entirely consistent with the RNA-seq data, the differences in gene transcription of several genes between subtypes further explained the heterogeneity of PD symptoms probably. For example, Isl1 in the TD subtype was significantly lower than in Mix (Fig. 3d), Gbx2 was up-regulated specifically in the PIGD subtype (Fig. 3h), and Lmx1a, Wnt9b were most significantly down-regulated in the Mix subtype (Fig. 3e, f).

### Subtypes of PD differ in neurotransmitter anabolic pathway and fatty acids

**Fig. 4:**
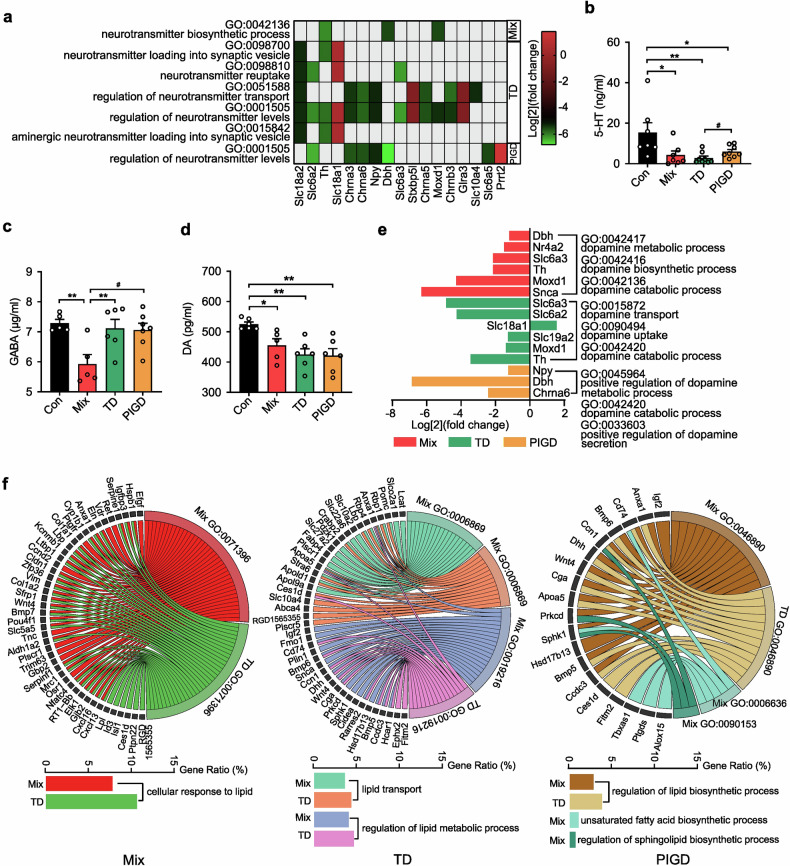
Analysis of neurotransmitter anabolic pathway and fatty acids in different subtypes of PD rats. **a**, **e** The biological function of neurotransmitter and dopamine in different subtype of PD rats. **b**–**d** 5-HT, GABA and DA concentration in SN. **f** The biological function of lipid and fatty in Mix, TD, and PIGD subtypes of PD rats. Data described are Mean ± SEM, n = 3-6. *p < 0.05, **p < 0.01 and ***p < 0.001 vs. control, ^#^p < 0.05 vs. other subtype

Neurotransmitters regulate physiological functions by transmitting various messages. The transmitter-related biological process (BP) suggests that there are differences in neurotransmitter levels between subtypes (Fig. 4a). Notably, the level of 5-HT in the SN of TD subtype rats was significantly lower than that in PIGD (Fig. 4b), and only the GABA concentration in the Mix subtypes was down-regulated (Fig. 4c), which may be one of the important downstream mechanisms for the occurrence of Mix subtypes. Additionally, dopamine (DA), a major central nervous system (CNS) neuro-messenger, was significantly down-regulated in the brain of PD rats (Fig. 4d). Although there was no difference in DA concentration among different PD subtypes, which is consistent with concentration serum levels in of PD patients, the three subtypes shows different changes in biological functions related to dopamine anabolism as analyzed by GO enrichment. As shown in Fig. 4e, the metabolism, synthesis and decomposition of DA were all dysregulated in rats with Mix PD. In contrast, the DEGs associated with the TD subtype were primarily involved in the transport and absorption of DA. The decrease of dopamine in PIGD subtype was mainly related to metabolism and secretion. Further analysis of BP for each group indicated that both the Mix and TD subtypes exhibited dysregulation in cellular response to lipids, regulation of lipid biosynthetic processes, lipid transport and regulation of lipid metabolic processes, although the DEGs were not identical (Fig. 4f). Regulations of the biosynthetic process for unsaturated fatty acids and sphingolipid were also altered between the Mix subtype and the control group. Furthermore, preliminary RNA-seq analysis did not reveal any lipid or fatty acid abnormalities in the PIGD subtype. Overall, the obtained DEGs inferred that the imbalance of lipids and fatty acids may be a significant etiology and pathogenesis factor contributing to tremor symptoms in patients with PD.

### Potential therapeutic effects of PD treatment candidates on subtypes

**Fig. 5:**
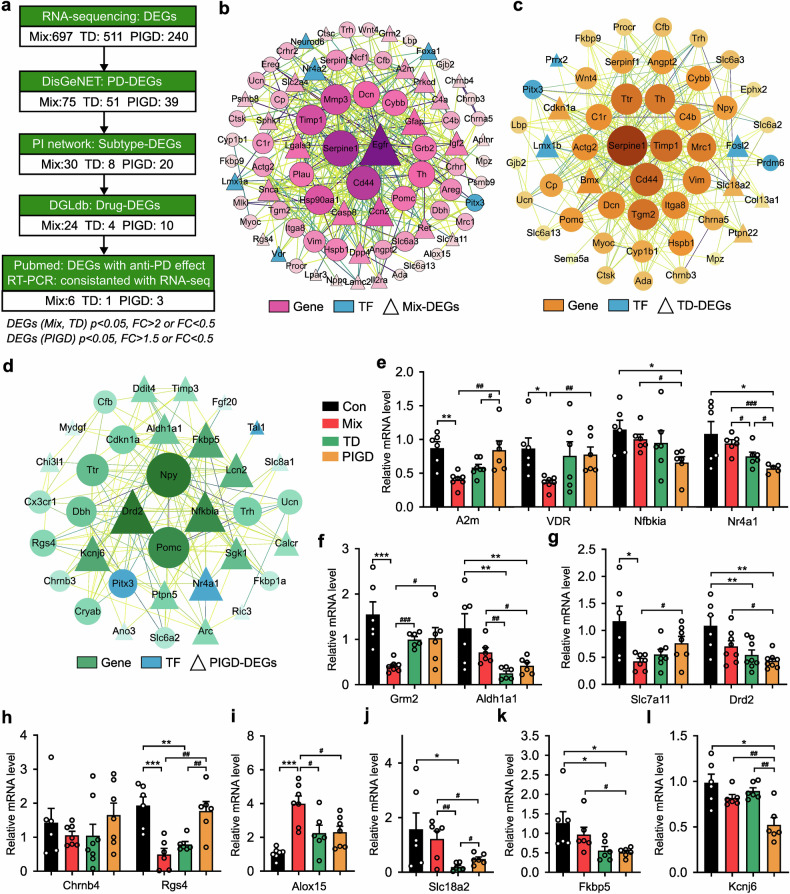
Potential therapeutic effects of PD treatment candidates on subtypes. **a** The flowchart illustrating the sequential steps involved in the search and selection process used in systematic data retrieval. **b**–**d** PPI network of Mix (**b**), TD (**c**) and PIGD (**d**) subtypes. **e**–**l** Relative mRNA levels of A2m, Chrnb4, Grm2, Rgs4, Slc7a11, VDR, Alox15, Slc18a2, Aldh1a1, Drd2, Fkbp5, Kcnj6, Nfbkia and Nr4a1 detected by RT-PCR. Data described are Mean ± SEM, n = 3–7. *p < 0.05, **p < 0.01 and ***p < 0.001 vs. control, ^#^p < 0.05 vs. other subtype

In order to find possible drug repursoing candidate genes, the functional connectivity of PD-associated DEGs in each subtype was examined using network analysis (Fig. 5a). As shown in Fig. 5b, 30 genes (e.g., EGFR, Casp8, Alox15 and Grm2) were found to be significantly up- or down-regulated exclusively in the Mix PD, thereby identifying them as candidate therapeutic targets for this subtype. Similarly, 8 and 25 candidate genes were identified for the TD (Fig. 5c) and PIGD (Fig. 5d) respectively. The DEGs were further queried in the Drug Gene Interaction Database (DGIdb), a data mining platform for investigating the druggable genome for personalized treatment, to identify their interacting compounds.^23^ As shown in Fig. 5e–l, genes without reported effective PD therapeutic drugs were excluded, as well as genes that were inconsistent with RT-PCR verification results (Chrnb4, Aldh1a1, Drd2, Fkbp5). The final group of candidate drugs (n = 16) was mapped to A2m, Alox15, Grm2, Rgs4, Slc7a11, and VDR genes for the treatment of the Mix subtype of PD. Amphetamine and LOBELINE were predicted to treat patients with the TD subtype of PD by up-regulating SLC18A2. Another group of drugs (n = 7) was suggested for the treatment of PIGD subtype based on the targets including Kcnj6, Nfkbia and Nr4a1 (Supplementary Table 1).

### Effectiveness of baicalein on different PD subtypes

**Fig. 6:**
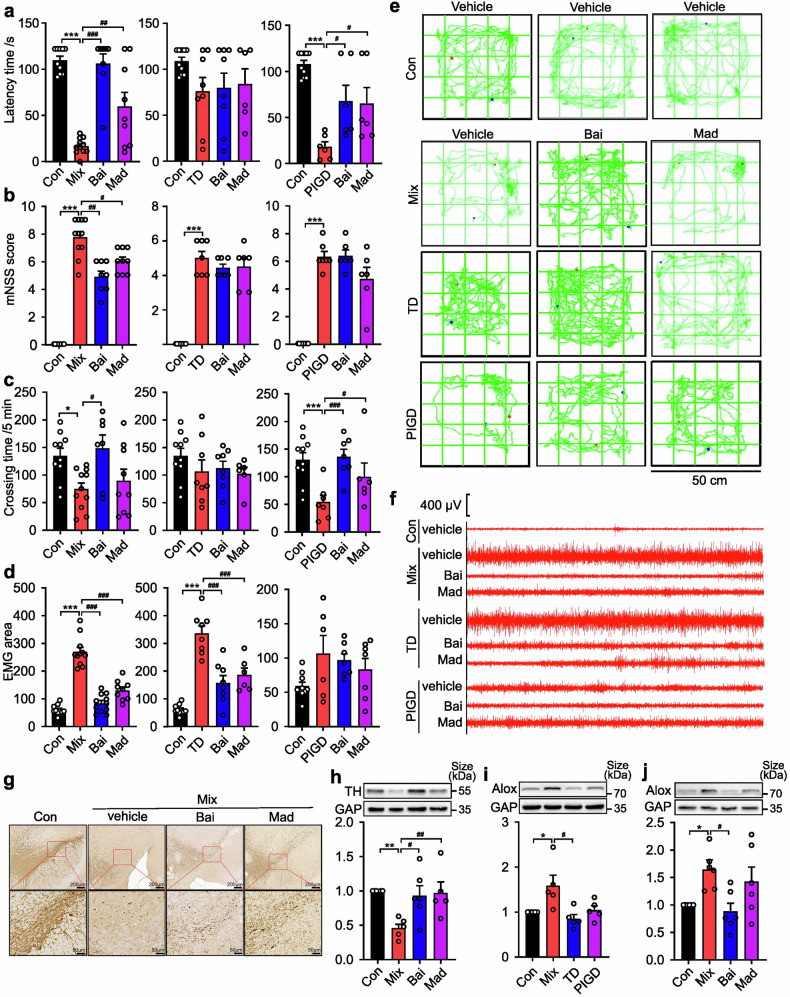
Effectiveness of baicalein (Bai) and madopar (Mad) on different PD subtypes of rats. **a** Rotarod test. **b** Neurological function test. **c** Open field test. **d** Electromyography. **e** The movement routes. **f** Segments of tremor monitor activity profiles. **g** Representative images of TH immunohistochemical staining in SN of rats. **h**–**j** Representative image of TH, Alox15 expression in the lesioned SN. Data described are Mean ± SEM, n = 3-12. *p < 0.05, **p < 0.01 and ***p < 0.001 vs. control, ^#^p < 0.05, ^##^p < 0.01 and ^###^p < 0.001 vs. other subtype

A growing body of evidence supports the hypothesis that baicalein exerts neuroprotective effects in PD due to its anti-apoptotic, pro-differentiation, and anti-oxidant properties.^24–26^ Based on the differential expression of Alox15 in the SN of different subtypes of PD rats, we further reported that baicalein, an Alox15 inhibitor,^27,28^ exhibited the most pronounced therapeutic effect on the Mix subtype of PD, especially in alleviating motor and tremor symptoms. As shown in Fig. 6a, baicalein increased the latency time of Mix rats by 281.6% (p < 0.0001), which was obviously greater than that of the PIGD group (148.5%, p = 0.041). Among rats with the Mix PD, a significant change in mNSS score was observed (p < 0.0001) (Fig. 6b). In addition, the Mix rats showed greater mean improvement in spontaneous locomotor activity following treatment with baicalein (p = 0.0135) compared to madopar (p = 0.8559) (Fig. 6c, e). EMG measurements indicated higher responsiveness to baicalein in the Mix group (71.1%, p < 0.0001) compared to the TD group (53.9%, p < 0.0001) (Fig. 6d, f). Furthermore, baicalein treatment (200 mg/kg) increased TH-positive neurons in the SN (Fig. 6g, h), and caused a significantly reduction in the expression of Alox15 (Fig. 6j), which was found to be overexpressed exclusively in the SN of Mix rats (Fig. 6i). As shown in Supplementary Fig. 2, baicalein treatment ameliorated the serum levels of the UA and NfL of the Mix rats, while the treatment effect was not significant in the other subtypes. Our findings confirmed previous studies indicating that regulating Alox15 expression has a specific effect on improving symptoms in Mix PD subjects.

## Discussion

Disease subtyping is essential to address PD heterogeneity in clinical symptoms to enhance both research and management strategies for developing disease-modifying treatments.^29^ In this research, we provided a comprehensive methodology to subtyping PD rats to address the challenges associated with subtype-specific mechanisms in PD research. Our findings indicated that baicalein, an Alox15 inhibitor, exhibits a significantly therapeutic effect on Mix rats. Increasing evidence suggests that Parkinson subtypes can be identified with cluster analyses, which employs a hypothesis-free, data-driven methodology.^30,31^ Similarly, we classified PD rats with 6-OHDA injury into Mix, TD, and PIGD subtypes based on a comprehensive battery of assessments, including rotarod test, open field test, neurological function test, and electromyography. This multifaceted approach prevents missing changes in motor performance due to the transience and volatility of a single behavioral indicator. In clinical studies, patients with PD are categorized into TD type, PIGD type, and indeterminate type (also called Mix type in some studies) based on the ratio of the mean tremor score to the mean motor retardation score. Clinically indeterminate PD is characterized by a ratio of 0.9-1.15, including patients with high tremor and high bradykinesia scores, as well as those with low scores for both. This subtype is considered transitional subtype of TD and PIGD types.^32^ Consequently, clinical studies have mostly focused on the TD and PIGD subtypes, resulting in a relative paucity of studies investigating the mechanisms underlying indeterminate PD.^3,33^ However, mechanistic studies suggest that the Mix subtype, characterized by high tremor and high bradykinesia scores, may not be transitional but rather a distinct subtype with specific pathological or molecular mechanisms crucial for PD research. Therefore, in the present study, PD rats and clinical patients with only high tremor and high bradykinesia symptoms were selected as the Mix subtype for mechanistic studies.

Despite limited clinical samples, numerous studies on serum indexes and neuroimaging characteristics of patients with different motor subtypes of PD^21,34,35^ provided a basis for examining the applicability of animal models and evaluating clinical consistency. Accordingly, the present study also detected the serum UA and NfL levels as well as alterations in brain autonomic activity in rats with different subtypes of PD. The findings revealed that over 70% of the metabolites detected in the serum of PD rats and clinical patients were altered concordantly. It confirmed the scientific validity and applicability of the rat model of PD subtypes in terms of biochemical indexes, cerebral neural circuits, and metabolites, which can be used for more extensive preclinical studies. Typical pathology of PD involves the degeneration of dopaminergic neurons and the formation of Lewy bodies.^36,37^ However, it is important to note that rats with different subtypes of PD do not show differential to dopamine neuron damages, but exhibit different abnormal aggregations of α-synuclein. To further explore the mechanism of the heterogeneity of PD, conducted RNA-seq analysis on the substantia nigra of rats. Our observations revealed that different subtypes were characterized by specific gene expression alteration compared to healthy rats, further demonstrating that PD patients with different symptoms represented three distinct subpopulations based on the disease. We found that the dysdifferentiation and apoptosis of CNS neurons lead to dyskinesia in PD rats, potentially explaining the poorer prognosis of PIGD patients.^38–42^ However, the dysfunction on PNS, particularly in myelin formation, combined with abnormal lipids and fatty acid metabolism, was found to induce tremors in PD rats (TD). Additionally, Neurons of the Mix subtype showed damage in both systems, influenced by ferroptosis, necroptosis, and disturbances in the apelin signaling pathway. And as the main inhibitory neurotransmitter in the brain, the reduction of GABA levels in SN also indicates that the pathogenesis of Mix PD may be related to GABAergic neuronal loss or dysfunction.^43^

Relating genetic variants to drug response has significant implications for choosing therapies for PD subtypes. In this study, we performed a drug-repurposing investigation utilizing key genes that regulate the expression profiles of Mix, TD and PIGD subtypes, identifying 25 drugs with known anti-PD effects. The occurrence of PD is mainly attributed to the degeneration of dopaminergic neurons, which is precipitated by lesions in the midbrain substantia nigra, thereby diminishing dopamine synthesis. Hence, the madopar, which supplements the insufficient neurotransmitter dopamine, is the major treatment for PD patients and was the positive drug in this study.^44^ In addition, the greater efficacy of baicalein as an Alox15 inhibitor on motor and tremor performance in Mix subtype of PD suggests that these targets deserve attention and further investigation. The process is in accordance with the goals of precision medicine, which is to improve diagnostic accuracy by identifying disease subtypes, ultimately assisting in the selection of specific treatments for well-defined individuals.

Despite its contributions, there were certain limitations to this study. Firstly, cluster analysis is constrained by the number of variables assessed and selected, as well as the number of clusters investigated, even though some clinician-scientists support it as the preferred method.^45^ Secondly, the gene-targeting medications database was not comprehensive, resulting in the omission of certain gene targets due to the lack of reported drugs with therapeutic effects for PD. Finally, given the sample size and the lack of evidence for protein function, the result of our analysis of associations between genomic characteristics and the onset of PD subtypes should be interpreted with caution and will require further research in future cohorts.

In summary, we provided a comprehensive genomic analysis of three subtypes of PD rats, categorized according to motor heterogeneity, and have provided detailed pathological annotations. It is our aspiration that these data will contribute to the design of future clinical trials aimed at identifying targeted therapies, thereby facilitating personalized treatment approaches.

## Materials and methods

### Drugs and reagents

6-OHDA hydrobromide and apomorphine hydrochloride were purchased from Sigma-Aldrich, USA. Antibody against Alox15 was purchased from Abcam, USA. Antibodies against TH, β-actin and GAPDH were purchased from Proteintech, China. Goat anti-rabbit/mouse antibodies were from CWBiotech, China. Trizol was from Invitrogen Life Technologies, USA. NEBNext® Ultra™ Directional RNA Library Prep Kit for Illumina® was purchased from NEB, USA. Uric acid (UA) test kit was purchased from Biosino Biotechnolgy and Science Inc., China. The enzyme-linked immunosorbent assay (ELISA) kit for DA was purchased from Jiangsu Meimian Industrial Co., Ltd, China. ELISA kits for 5-Hydroxytryptamine (5-HT) and NfL were purchased from CUSABIO, China. ELISA kit for Gamma-aminobutyric (GABA) was ImmuSmol, France. HiScript III All-in-one RT SuperMix Perfect for qPCR and 2X Taq Pro Universal SYBR qPCR master mix were purchased from Vazyme Biotech, China.

### Experimental animals

Male Sprague–Dawley rats (190–210 g, certificate NO. SCXK (Jing) 2016-0011) were supplied by Beijing Vital River Laboratory Animal Technology Co., Ltd. In an SPF setting with a 12/12 light-dark cycle (22–25 °C, 60–70% humidity), the animals had free access to water and food. All experiments were performed in accordance with the National Institutes of Health Guidelines for the Care and Use of Laboratory Animals. And protocols were approved by the Animal Care and Use Committee of the Institute of Materia Medical, Chinese Academy of Medical Sciences.

### Patients selection

A total of 17 patients with a clinical diagnosis of PD were evaluated at the Movement Disorders Clinic at the Beijing Tiantan Hospital, Capital Medical University. Healthy controls (HC) were selected among patients’ caregivers. This study was approved by the ethics committee of Beijing Tiantan Hospital, Capital Medical University. Informed consent was obtained from all volunteers. At baseline, standardized neurological examination was performed, including the Movement Disorder Society- Unified Parkinson Disease Rating Scale (MDS-UPDRS)^46^ and Hoehn and Yahr stage (H&Y) assessment.^47^

### Classification of PD patients into Mix, TD, and PIGD subtypes

All 17 patients were divided into Mix group, TD group, and PIGD group according to the mean tremor score and PIGD score. The mean tremor score was the sum of MDS-UPDRS item 2.10, 3.15a, 3.15b, 3.16a, 3.16b, 3.17a, 3.17b, 3.17c, 3.17d, 3.17e, 3.18 divided by 11, whereas the mean PIGD score was the sum of MDS-UPDRS item 2.12, 2.13, 3.10, 3.11, 3.12 divided by 5. Accurately, 5 patients with ratio ≤0.9 were placed in PIGD group, and 6 patients with the ratio ≥1.15 were placed in the TD group. Six patients with both mean tremor score and PIGD score greater than 0.8 were categorized as Mix group, which is different from previous studies.^32^

### Surgery

Rats were anesthetized with 3% sodium pentobarbital (45 mg/kg i.p.). 6-OHDA lesions were performed as previously described in our laboratory.^48^ Briefly, 4 μl of freshly prepared 6-OHDA solution (4 μg/ul, dissolved in 0.04% ascorbate saline) was injected unilaterally in a stereotaxic manner (0.5 μL/min) in the left medial forebrain bundle (MFB), the deposit at the following coordinate according to the atlas of Paxinos and Watson (1986) (in mm relative to bregma and the surface of the dura mater): anterior (A)= −4.0, lateral (L) = 1.65, ventral (V)= −8.0. The sham-lesioned rats received only vehicle at the same coordinates. Behavioral tests were implemented at 3 weeks after surgery.

### Administration of baicalein

Previous studies showed that baicalein at doses of 100, 200, and 400 mg/kg could significantly suppress tremor in PD rats. Thus, 200 mg/kg was determined as the dosage for examining the effect of baicalein in different subtypes of PD rats. Following the subtyping, the rats with each subtype were randomly allocated into 3 groups: models (CMC-Na i.g.), baicalein (200 mg/kg/d i.g.) and madopar (50 mg/kg/d i.g.). All the rats were treated as described for 4 weeks.

### Rotational behavior test

After administration of apomorphine (0.5 mg/kg, s.c.), the rats were placed in individual transparent containers with a diameter of 40 cm. After the rats habituated to the surroundings for 10 min, the frequency of rats making 360° turns in the contralateral direction of the lesion in 30 min was counted.

### Rotarod test

Motor performance of 6-OHDA-induced rats on a moving rod was measured by the DXP-3 rotarod system (Institute of Materia Medica, Chinese Academy of Medical Sciences and Peking Union Medical College, Beijing, China).^25^ From the rat was placed on the rotating rod at a rotation speed of 6 s/rpm, the time was counted until it fell off the rod within 120 s, which was expressed as latency time. Before the surgery, rats were treated to learn to run on the rotarod rod for 3 days.

### Open field test

Spontaneous locomotor activity of rats was measured by the open field test. Rats were placed for 5 min in the central square of a box (50 cm × 50 cm × 50 cm) whose bottom was divided into 25 equal squares.^49^ The number of squares crossed was counted by SuperMaze software (Shanghai XinRuan Information Technology Co., Ltd, Shanghai, China). The open field apparatus was cleaned before each test.

### Neurological function test

Neurological function of rats was tested by an investigator without any prior knowledge, i.e., blind, to group allocation conditions. Based on the behavioral characteristics of 6-OHDA-induced rats, Longa sores and neurological severity scores (NSS) were modified. Excluding reflexes, neurological functions including motor (4 points), sensory (2 points), and balance (6 points), scores were graded on a numeric scale from 0 to 12,^50^ with higher scores denoting greater severity.

### Electromyography recording

Tremor in 6-OHDA-induced rats was recorded by BL-420S (Tme, Chengdu, China).^24^ The double recording electrodes were inserted into the gluteal muscle of the rats, and the reference electrode was grounded. The bioinformation collector BL-420S was used to detect electromyography (EMG) signals on the hind limb of awake rats. Stimulus signal was 1 mV, range was 1 mV, high-pass filtering was 0.001 s, low-pass filtering was 10 kHz.

### Immunohistochemical staining

Rats were intracardially perfused with 4% paraformaldehyde. The brains were immediately isolated. After paraffin-embedded tissues were blocked by serum, they were incubated with the primary antibodies (TH and α-syn) overnight at 4 °C and secondary antibody at 25 °C for 2 h. Then, the immunohistochemical sections were co-incubated with 3,3’-diami-nobenzidine peroxide (DAB) and counterstained with hematoxylin. And the immunofluorescence sections were incubated with 4’,6-diamidino-2-phenylindole (DAPI) solution for 10 min at room time. Nico Eclipse Ti-SR (Nikon, Tokyo, Japan) was used to capture the images. Average number of positive cells quantitated by Image-Pro Plus 6.0 was used to represent cell density.

### Transcriptome analysis of DEGs

Since the results of TH immunohistochemical staining showed different degrees of damage to dopaminergic neurons in the SN in different subtypes of PD rats, total RNA of the SN was extracted using Trizol reagent (Invitrogen Life Technologies). The concentration, quality and integrity were determined by a NanoDrop spectrophotometer (Thermo Scientific). cDNA library was established by NEBNext® Ultra™ Directional RNA Library Prep Kit for Illumina®. After cDNA libraries were purified (AMPure XP system, Beckman Coulter, Brea, FL, USA) and quantified (Bioanalyzer 2100 system, Agilent, Palo Alto, CA, USA), cDNA was sequenced on a NovaSeq 6000 platform (Illumina, San Diego, CA, USA). The reference genome and gene annotation files were downloaded from genome website. The filtered reads were mapped to the reference genome using HISAT2 v2.0.5. DESeq2 software was used to evaluate DEGs under the following screening conditions: expression difference multiple |log2FoldChange | > 1, significant p-value ≤ 0.05.

### LC-MS analysis of metabolite profiles

Considering the easy availability of clinical serum samples, serum from rats was also selected for the following experiments. Metabolomics analysis was selected in rats and patients in the control group (n = 6), Mix group (n = 6), TD group (n = 5–6) and PIGD group (n = 6). Serum were extracted at 100 μL per 500 μL methanol. After centrifugation at 4 °C (14,000 rpm, 20 min), the supernatant of each sample was examined. 1H-NMR spectra from a Bruker 600 MHz Avance III NMR spectrometer were used MestReNova software (Mestrelab Research, Santiago de Compostella, Spain) to obtain the Fourier transform. Then signal peaks in the spectra were matched to specific metabolites using the documented literature as well as the NMR database. Finally, the different metabolites were screened to investigate metabolic pathways by MetaboAnalyst 5.0.

### Assay of NfL, UA, and neurotransmitters content

The content of UA was detected by the automatic biochemical analyzer (Toshiba Accute TBA-40FR, Toshiba Corporation, Tokyo, Japan) according to the manufacturer’s instructions. Using a microplate reader, the content of neurotransmitters (5-HT, DA, GABA) in the lesioned SN and the content of NfL in the serum were detected following the manufacturer’s ELISA kit guidelines.

### Quantitative real-time PCR

As described in previous research,^51^ total RNA was extracted with Trizol regent from lesioned SN. Using anMonScript™ 5 × RTIII all-in-one mix, the complementary DNA was reverse transcribed following RNA quantification. Quantitative RT-PCR was performed using 2X Taq Pro Universal SYBR qPCR master mix on aa FQD96 RT-PCR Detection System (Bioer Technology Co., Ltd., Hangzhou, China) for 45 cycles with temperature cycling and denaturation according to instructions of the manufacturer. The results were normalized with β-actin, and Supplementary Table 2 shows gene-specific primer pairs.

### Western blot

Rat brain tissues were lysed according to the protocol using RIPA buffer. Equal amounts of total protein were separated by 12% SDS-PAGE and transferred onto PVDF membranes. After blocking with 5% skim milk in TBST, the membranes were washed and incubated with antibodies. Finally, the bands were visualized by Tanon 4600 Imaging System (Tanon Technology Co., Ltd, Beijing, China) of super ECL (Applygen Technologies Inc. Beijing, China).

### Statistical analysis

The quantitative experimental data were calculated as means ± standard deviation (SEM), representative of at least three independent experiments. The differences between control group and subtypes of PD groups were determined using one-way ANOVA, and the difference between each two groups of subtypes was tested by Student’s *t*-test. P < 0.05 were considered statistically significant.

## Supplementary information

Sigtrans_Supplementary_Materials

Western Blots

## Data Availability

All data generated or analyzed during this study are available from the corresponding authors upon reasonable request.
