# Supplementary material for: Identifying genetic targets in clinical subtypes of Parkinson’s disease for optimizing pharmacological treatment strategies
Source: Signal Transduct Target Ther. 2024 Nov 18;9:320. doi: 10.1038/s41392-024-02020-x (PMC11570617; doi:10.1038/s41392-024-02020-x)
Supplement: Supplementary file 1 — Sigtrans_Supplementary_Materials [file 41392_2024_2020_MOESM1_ESM.docx]

Supplementary Materials for

Identifying genetic targets in clinical subtypes of Parkinson’s Disease for optimizing pharmacological treatment strategies: a rat study

Dewen Kong, Cao Li, LingYan Ma, Lida Du, Nan Jiang, Xiaoyue Zhao, Sen Zhang, Zhigang Zhao, Lianhua Fang, Guanhua Du.

Correspondence to: [1022zzg@sina.com](mailto:1022zzg@sina.com), [fanglh@imm.ac.cn](mailto:fanglh@imm.ac.cn) or [dugh@imm.ac.cn](mailto:dugh@imm.ac.cn)

**This PDF file includes:**

Materials and Methods

Figures. S1 to S2

Tables S1 to S3

Materials and Methods

**Rs-fMRI data acquisition and analysis**

Rats were anesthetized with isoflurane during resting state fMRI procedures. MR-imaging was carried out on a 7.0T PharmaScan 70/16 US system (Burker, Germany). The gradient echo-planar imaging (EPI) sequence and T2WI sequence were used to acquire functional and anatomical images respectively with the following settings: TR = 2000 ms, TE = 20 ms, flip angle = 90°, data matrix = 64×80, time points = 400, slice thickness = 0.65, slice number = 45. A series of conventional preprocessing steps were performed, including (1) discarding first 20 volumes and correcting slice-timing; (2) head-motion correction; (3) linear detrending and space normalization; (4) Gaussian smoothing for normal distribution; (5) Linear drift removal; (6) Regressing out white matter, cerebrospinal fluid and 24 motion parameters; (7) temporal filtering (band-pass 0.01–0.1 Hz). We used DPABI software to calculate the amplitude of low frequency fluctuations (ALFF) values, following by a two-sample t-test between Mix (or TD or PIGD) with control groups.


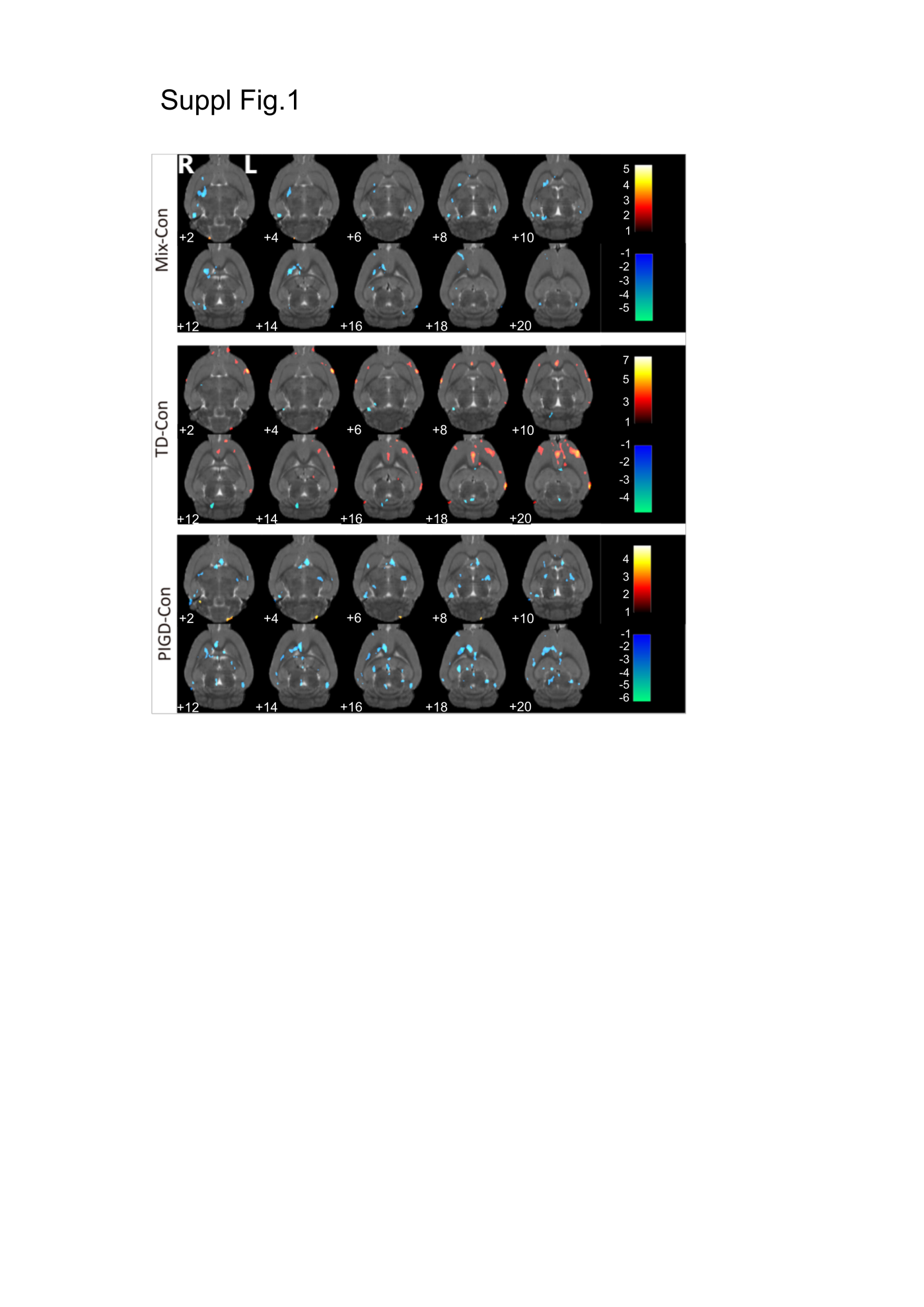


Figure. S1. Brain maps for between-group differences in ALFF values. The statistical threshold was set at P < 0.01 and cluster size >20 voxels, which corresponds to a corrected P < 0.01. T-score bars are shown on the right. Hot and cold colors indicate PD-related ALFF increases and decreases, respectively.


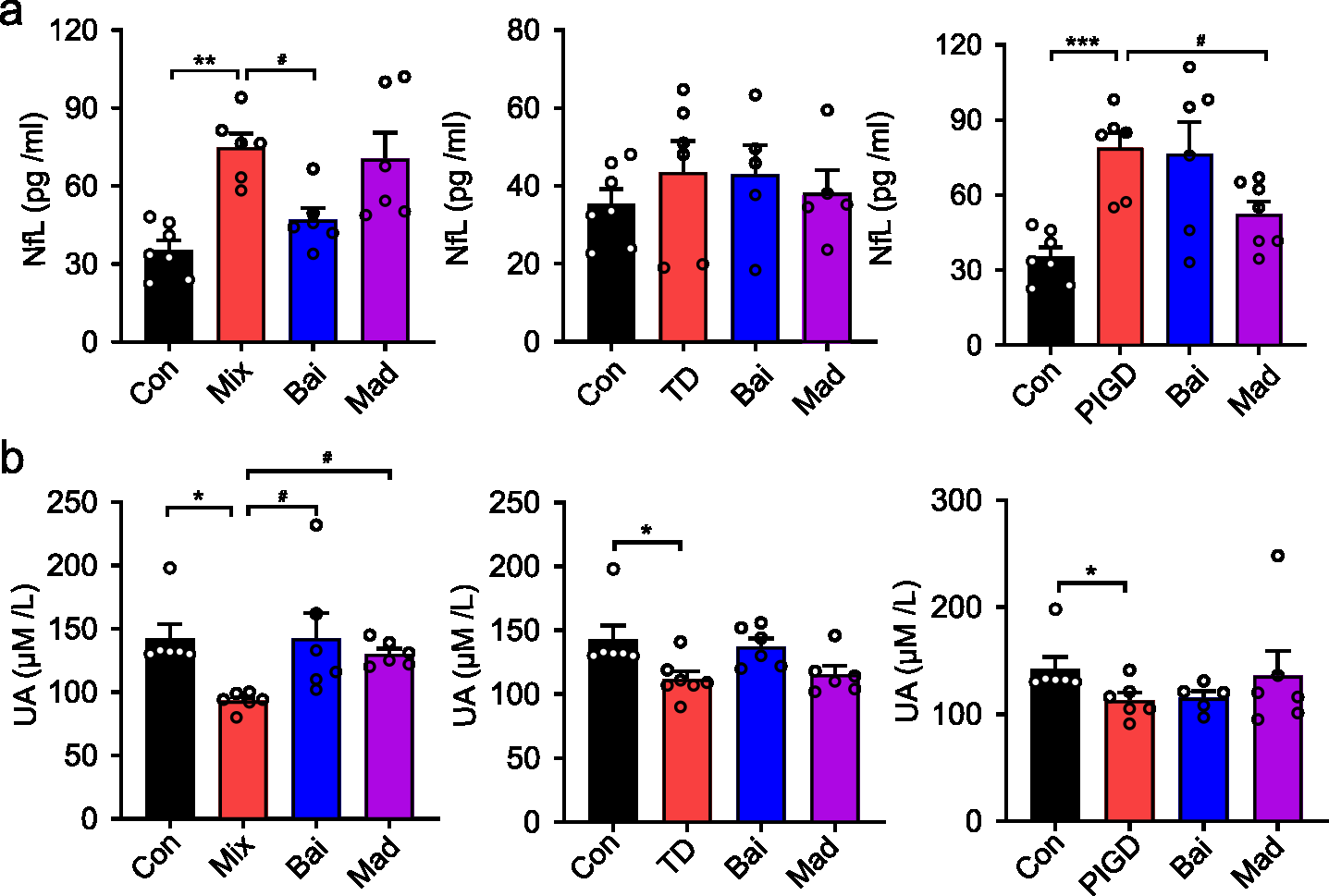


Figure. S2. Effectiveness of baicalein on different PD subtypes of NfL and UA concentration in serum. The data are presented as the mean ± SEM, n=6. *P < 0.05, ***P < 0.001 vs the control group, ^#^P < 0.05 vs one subtype group.

Table S1. Compounds and drugs obtained from the therapeutic gene targets.

| Subtype | Gene | Drugs | MaxPhase | | Structure | | PMID |
| --- | --- | --- | --- | --- | --- | --- | --- |
| Mix | A2m | [THROMBIN](https://dgidb.org/drugs/THROMBIN) | | Approved | |  | 15619541 2432677 |
| Mix | Alox15 | CHEMBL241813 | | Preclinical | 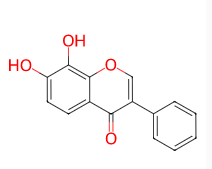 | | 17869117 |
| Mix | Alox15 | CHEMBL8328 | | Preclinical | 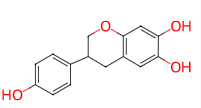 | | 17869117 |
| Mix | Alox15 | Baicalein | | Phase2 | 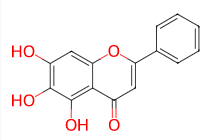 | | 28595877 36427096 34087694 34666128 28634902 35237220 36670874 30385133 |
| Mix | Alox15 | GERANIOL | | Preclinical | 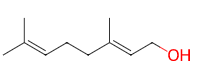 | | 27026137 35900613 30141137 23943375 |
| Mix | Alox15 | NEROLIDOL | | Preclinical | 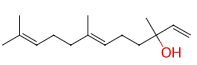 | | 27549180 |
| Mix | Alox15 | BISABOLOL | | Preclinical | 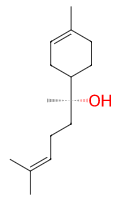 | | 33049992 |
| Mix | Alox15 | CITRONELLOL | | Preclinical | 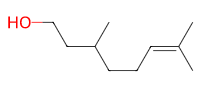 | | 36097250 36387498 |
| Mix | Grm2 | LY2979165 | | Phase 1 | 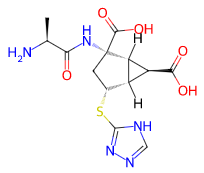 | | 32052375  33071070  29564482 |
| Mix | Grm2 | JNJ-40411813 | | Phase 2 | 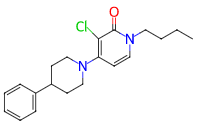 | | 25462291  25586401  25735992 |
| Mix | Grm2 | LY2969822 | | Phase 1 | 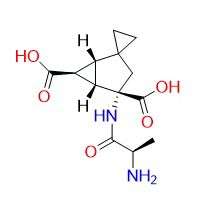 | | 28177520  31306647  30934533 |
| Mix | Grm2 | LY404039 | | Phase 1 | 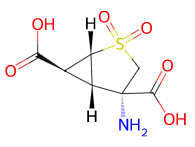 | | 32403118  32403118 |
| Mix | Rgs4 | LEUKOTRIENE D4 | | Preclinical | 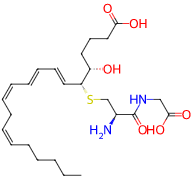 | | 36820885 |
| Mix | Slc7a11 | RILUZOLE | | Approved | 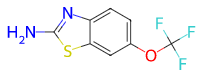 | | 22480308 19029129 11835434 10899284 20226190 12629173 |
| Mix | VDR | CALCIPOTRIOL | | Approved | 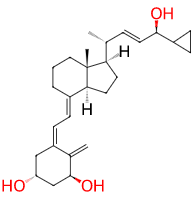 | | 28164279 17274004 21651652 10707958 17016423 |
| Mix | VDR | CALCITRIOL | | Approved | 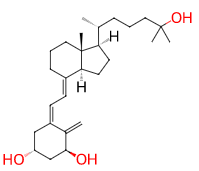 | | 36187296 34393753 24858239 22285943 15883634 |
| TD | SLC18A2 | Amphetamine | | Approved | 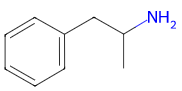 | | 25220836 34951464 30741691 16835371 |
| TD | SLC18A2 | LOBELINE | | Phase 2 | 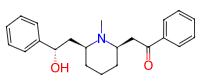 | | 24396408 17233532 |
| PIGD | Kcnj6 | FLUPIRTINE | | Approved | 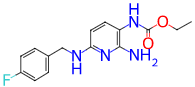 | | 12140664 9185829 8811503 |
| PIGD | Nfkbia | DIOSCIN | | Preclinical | 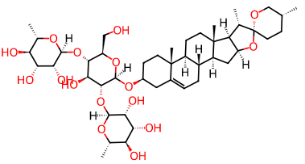 | | 35630630 23621869 |
| PIGD | Nfkbia | WEDELOLACTONE | | Preclinical | 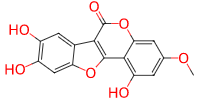 | | 32894501 22926226 |
| PIGD | Nr4a1 | CYTOSPORONE B | | Approved | 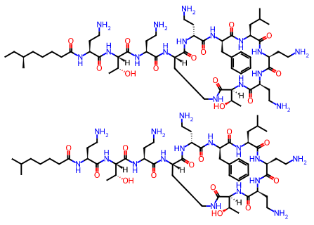 | | 32401747 27889907 18690216 |
| PIGD | Nr4a1 | LEVODOPA | | Approved | 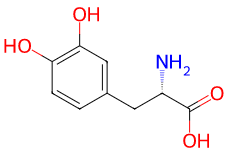 | | 25449210 18781671 16930409 15896973 |
| PIGD | Nr4a1 | ACETYLCYSTEINE | | Approved | 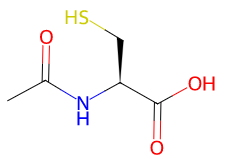 | | 30551603 24683506 8647183 |
| PIGD | Nr4a1 | NICOTINE | | Approved | 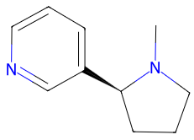 | | 18683238 28680589 1211143811979430 |

Table S2. Sequences of primers for real-time PCR. F: forward; R: reverse.

| Name | Primer sequence /F | Primer sequence /R |
| --- | --- | --- |
| A2m | GGCGAGGTTCTCCATCAACA | CAGTGTGCCAAGCCTCTACA |
| Alox15 | GGCTCAGGCTTGCTACTTCA | ATGGTGCAGGGTGCATTAGG |
| Chrnb4 | CACCAACGTGATTGTGCGTT | TCATAGGTCCAGGAGCGGAA |
| Dpp4 | CCCAGTTTAACGACACCGGA | CCTGCCTTTGGGTACGGAAT |
| Grm2 | AGTGGTGACATTGCGCTGTA | TGCGGGTCTTGAAGGCATAG |
| Rgs4 | GAGGAGACAAGCCGGAACAT | GCTGGAAGGATTGGTCAGGT |
| Slc7a11 | CTGGGCATGAGAAGGTGGTT | TATGCCCTTGGGGGAGATGA |
| Vdr | GCATCCAAAAGGTCATCGGC | TGATCACCTCAATGGCGCTT |
| Slc18a2 | GTCTCCACCTCCGAAAGCAT | GGACAGTGGTGTTAGCCACA |
| Aldh1a1 | AGGCTTTCCAGATTGGCTCC | GCAAAGACTTTCCCACCATTGA |
| Drd2 | CTGGCTGCCCTTCTTCATCA | TAGATGATGGGGTTGACGGC |
| Fkbp5 | CAGAGCAGGATGCCAAGGAA | TCCCATGGTCTGACTCTCGT |
| Kcnj6 | CAGGGTCATCACGGACAAGT | TCTCTGCCCTCTTCTTGGGT |
| Nfkbia | CCCAAGTACCCGGATACAGC | AGGGCAACTCATCTTCCGTG |
| Nr4a1 | CAGGTGTATGGCTGCTACCC | GGCTGGAAGTTGGGTGTAGG |
| Wnt2 | GTTCTTGAAACAAGAGTGCAAGTG | CCCATTGTACTTCCTCCAGAGATA |
| Lmx1a | CCTACGGTGCTGAACCTCTT | ACTCTGGACTGTAGGGGTCC |
| Wnt9b | AAGAGCGGCCTGAGAACAAC | ACTTGGCCAGTCTCACGAAA |
| Itgb4 | ATGGCCAACCGCTGTAAGAA | GCTCCTTGAACAGCTCGTCT |
| Prx | CAAGATGCCCCGTCTCAGTT | AGGTGGGCATTCGAAGTCTG |
| Ncmap | ACCTTGGTGCTGATCTTGCT | CTGCCATTGCTGTTTGGGTC |
| Isl1 | ACCTGCTTTGTTAGGGACGG | GTGGTACACCTTAGAGCGGG |
| Cldn1 | ACTGTGGATGTCCTGCGTTT | AAAGATTGCGATCAGCCCCA |
| Myoc | TGGAGGATTGACACGGTTGG | GGGAGCACATGGACCTTTGA |
| Dhh | TGTGCCTCTGCTCTACAAGC | GGTTGTAGTTGGGGACGAGG |
| Gabrb1 | CAACAGGGGCATATCCACGA | GTGTGGAGGGCATGTAGGTC |
| Gbx2 | TCAAACTCAGCGAGGTGCAA | CCGTCTTGGAATTGGCGTTG |
| Foxp2 | CCTCAAACCCCTACCACACC | AATGTCGCCTTCGTATGGCT |
| Ucn | GCGCACTCCTCTTGCTGTTA | AGGTGGAAGGTGAGGTCGA |
| Stra6 | GGCCCTCTTCAGCAATCTGT | CTTGATGTCTCCGGCTCTCC |
| Ntsr1 | CAAGGTGCTGGTGACTGCTA | CCCAGGTGGTAATGCACAGT |
| β-actin | CACGATGGAGGGGCCGGACTCATC | TAAAGACCTCTATGCCAACACAGT |

Table S3. Brain regions with decreased ALFF

| Brain regions | MNI coordinates | | | Ke | MAX_T |
| --- | --- | --- | --- | --- | --- |
|  | X | Y | Z |  |  |
| TD<Con |  |  |  |  |  |
| medulla oblongata-L | 1.9917 | 7.8027 | -0.3579 | 35 | 3.755 |
| thalamus_lateral nucleus group-L | 2.9523 | 6.4341 | -2.2779 | 33 | 4.2617 |
| striatum-L | 1.7256 | 7.9044 | 0.1221 | 24 | 3.5492 |
| cerebellum _cerebellar nucleus-L | 2.9523 | 6.2748 | -2.2779 | 21 | 4.3383 |
| visual cortex-R | -4.4289 | 4.5453 | -8.9979 | 19 | 4.7693 |
| olfactory cortex-R | -4.4223 | 4.5879 | -9.4779 | 18 | 4.4138 |
| poaterior lobe of cerebellum-L | 1.607 | 6.9976 | -1.3179 | 14 | 3.384 |
| PIGD<Con |  |  |  |  |  |
| anterior lobe of cerebellum-L | 0.653 | 4.1785 | 0.1221 | 33 | 5.3287 |
| Striatum-L | 4.9591 | 8.3024 | -2.2779 | 28 | 5.3477 |
| cingulate gyrus-L | 0.5146 | 4.3298 | 0.1221 | 22 | 5.7199 |
| Mix<Con |  |  |  |  |  |
| sensory cortex-R | -3.7189 | 4.6216 | -2.7579 | 142 | 4.0131 |
| Thalamus_lateral nucleus group-L | 1.897 | 7.5413 | -2.2779 | 84 | 4.9354 |
| corpus callosum-R | -3.5805 | 4.6296 | -2.7579 | 51 | 4.0294 |
| visual cortex-R | -5.5081 | 4.7416 | -8.5179 | 49 | 5.4089 |
| visual cortex-L | 4.7656 | 4.6247 | -7.0779 | 44 | 3.8893 |
| medulla oblongata-L | 1.9168 | 8.678 | -3.7179 | 36 | 5.7538 |
| poaterior lobe of cerebellum-R | -3.9101 | 4.4144 | 1.0821 | 35 | 3.6653 |
| medulla oblongata-R | -3.0376 | 8.6503 | -3.2379 | 32 | 3.8178 |
| hippocampus-R | -3.7123 | 4.6642 | -3.2379 | 32 | 4.1467 |
| pontine_tegmentum of pons-L | 2.0181 | 7.6899 | -2.2779 | 28 | 4.4558 |
| cerebellum _cerebellar nucleus-R | -2.919 | 6.1941 | -1.7979 | 27 | 3.9636 |
| poaterior lobe of cerebellum-L | 2.0181 | 7.389 | -2.2779 | 26 | 4.4922 |
| thalamus_lateral nucleus group-R | -3.031 | 8.2504 | -3.7179 | 21 | 3.4004 |
| hypothalamus_tuberal region-L | 1.785 | 8.571 | -4.1979 | 19 | 5.4456 |
| tegmentum of midbrain-L | 1.7916 | 8.6136 | -4.6779 | 19 | 5.0275 |
| temporal association cortex-R | -5.5081 | 5.0248 | -8.5179 | 19 | 4.9159 |
| temporal association cortex-R | 1.8904 | 7.0562 | -1.7979 | 18 | 4.5069 |
| septal area-R | -2.919 | 6.0525 | -1.7979 | 17 | 3.7203 |
| striatum-R | -5.0576 | 6.8384 | -2.2779 | 14 | 3.3639 |
| hippocampus-R | 1.2487 | 5.6195 | -4.1979 | 11 | 3.0147 |
