# Supplementary figures and images for: Identifying genetic targets in clinical subtypes of Parkinson’s disease for optimizing pharmacological treatment strategies
Source: Signal Transduct Target Ther. 2024 Nov 18;9:320. doi: 10.1038/s41392-024-02020-x (PMC11570617; doi:10.1038/s41392-024-02020-x)

Fig 6h

TH

1-1

GAPDH

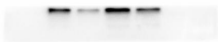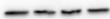

1-2

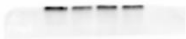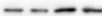

3-1

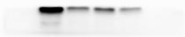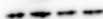

4-1

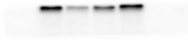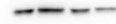

4-2

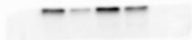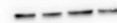

Fig 6i  
Alox15  
2-1

GAPDH

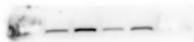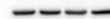

3-1

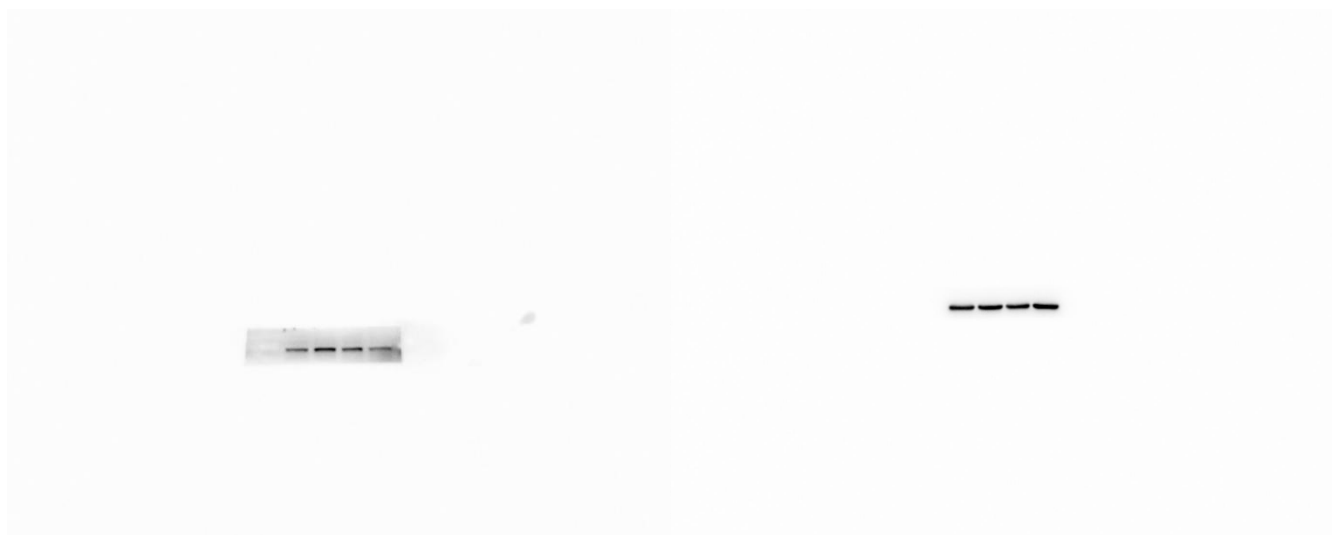

4-1

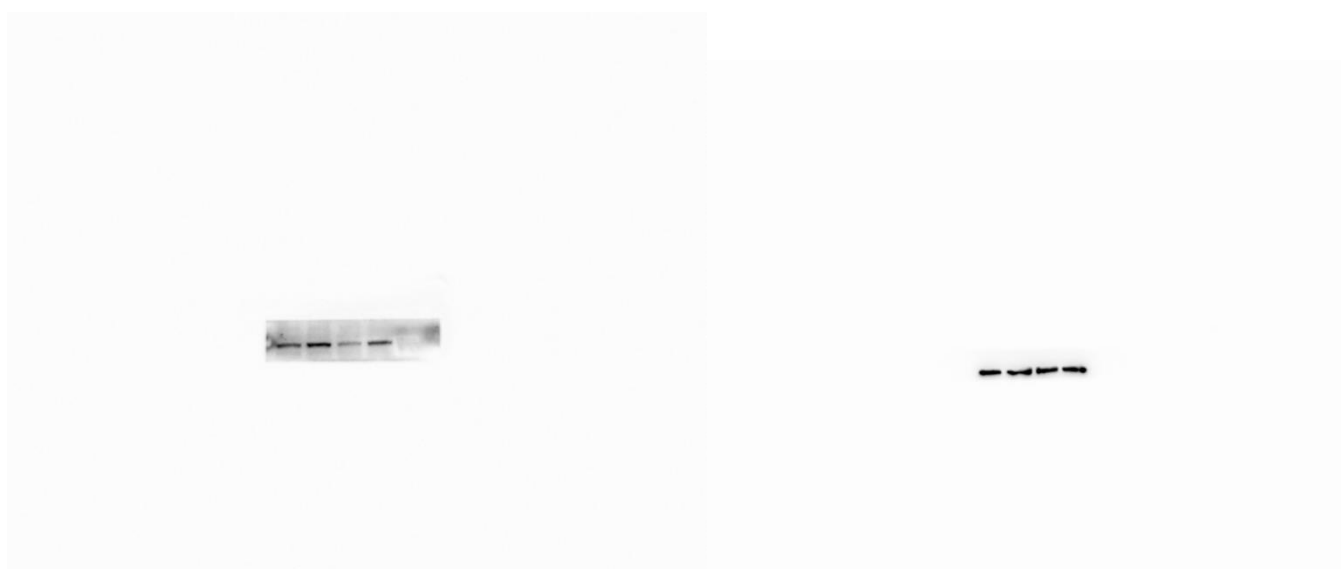

3

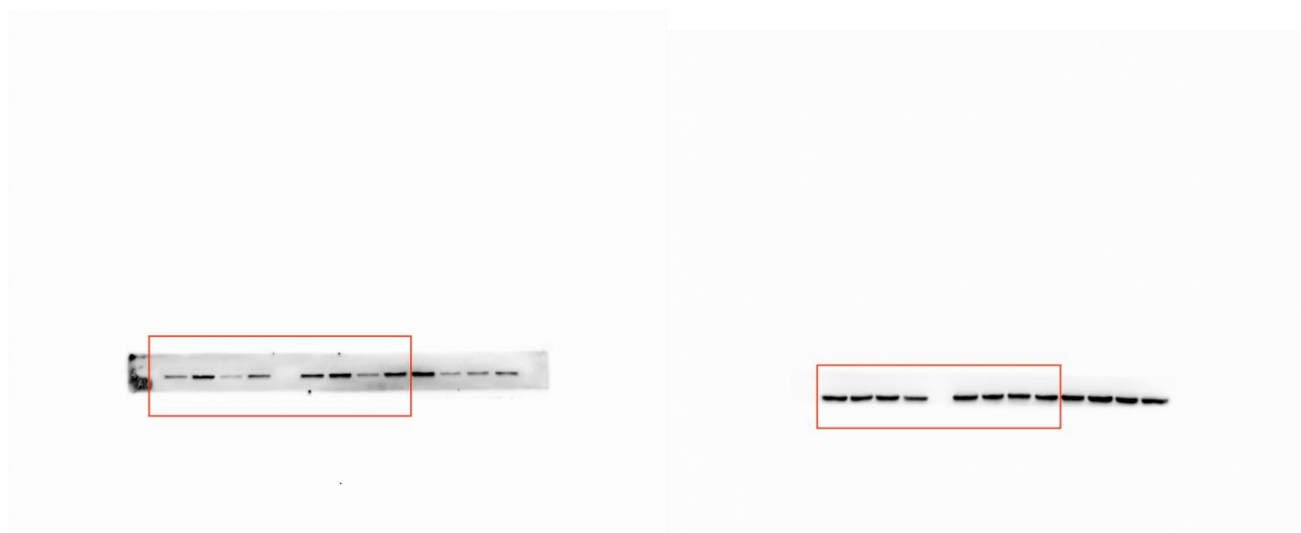

Fig 6j  
Alox15  
1-1

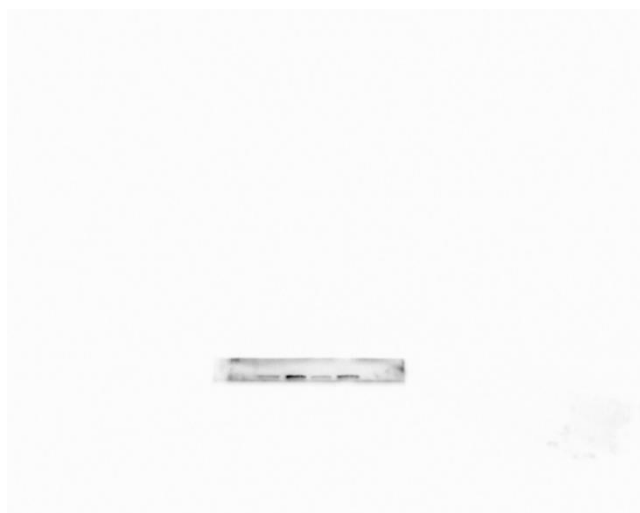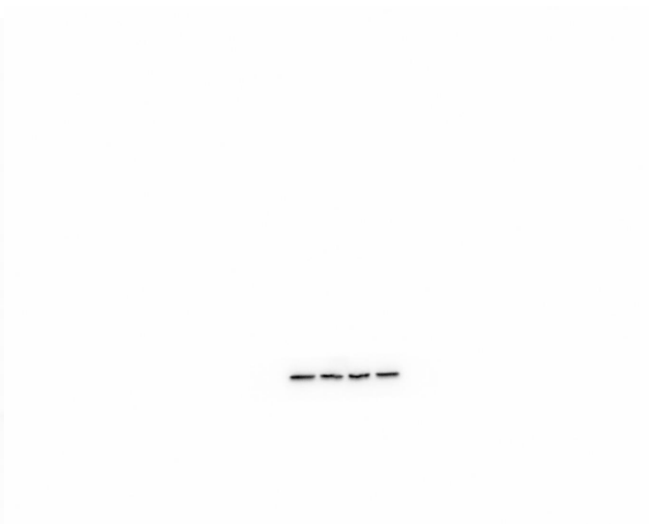

2-2

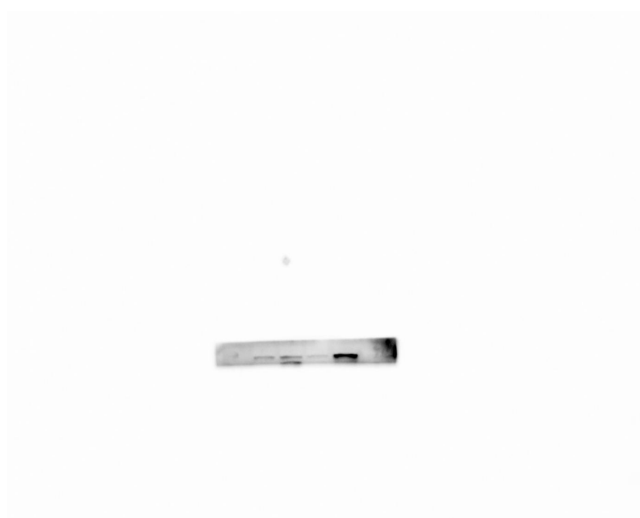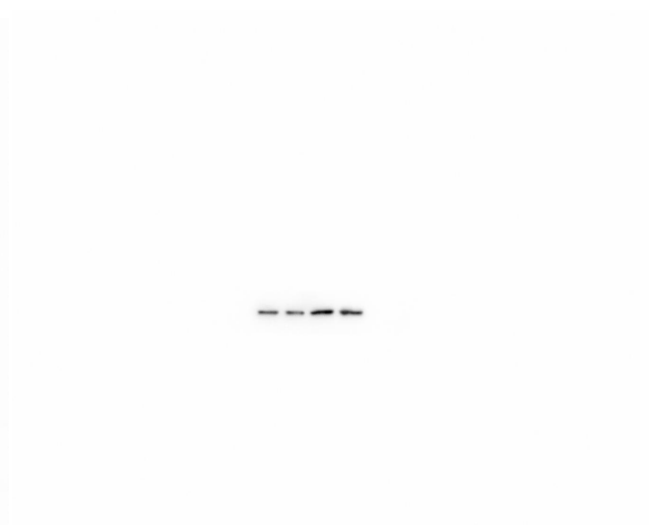

3-2

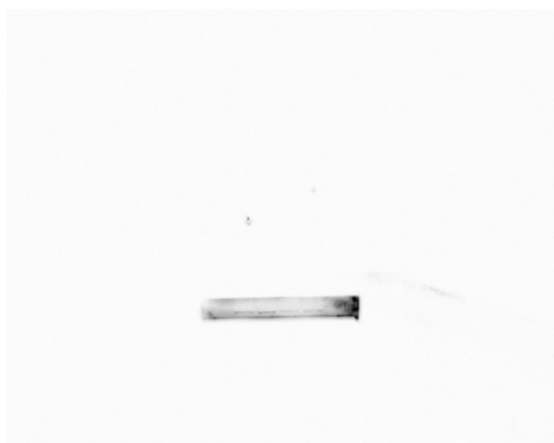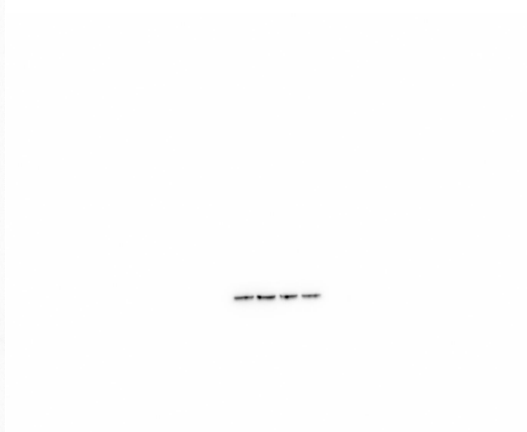

1-1

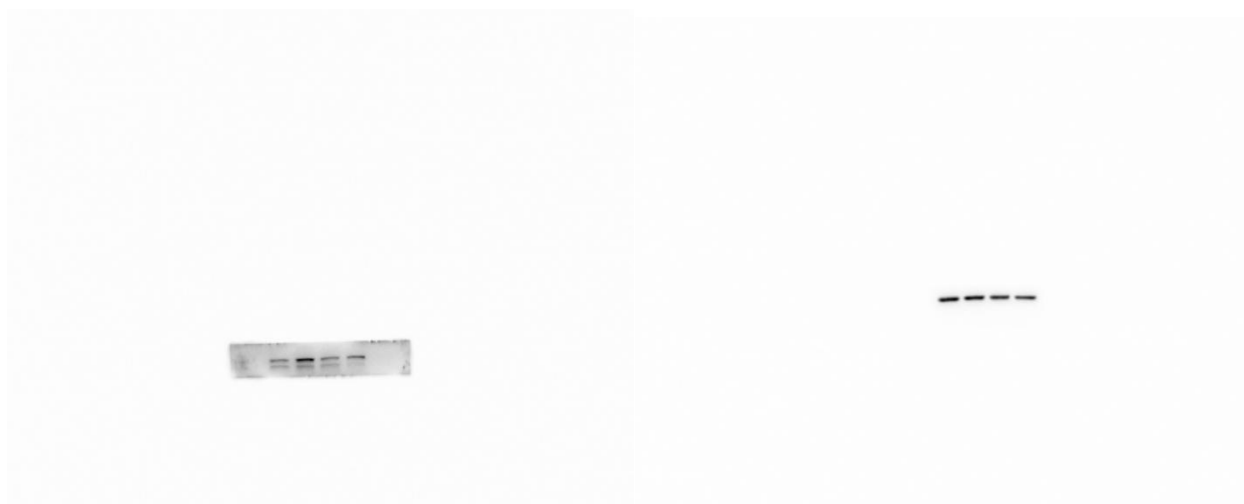

2-1

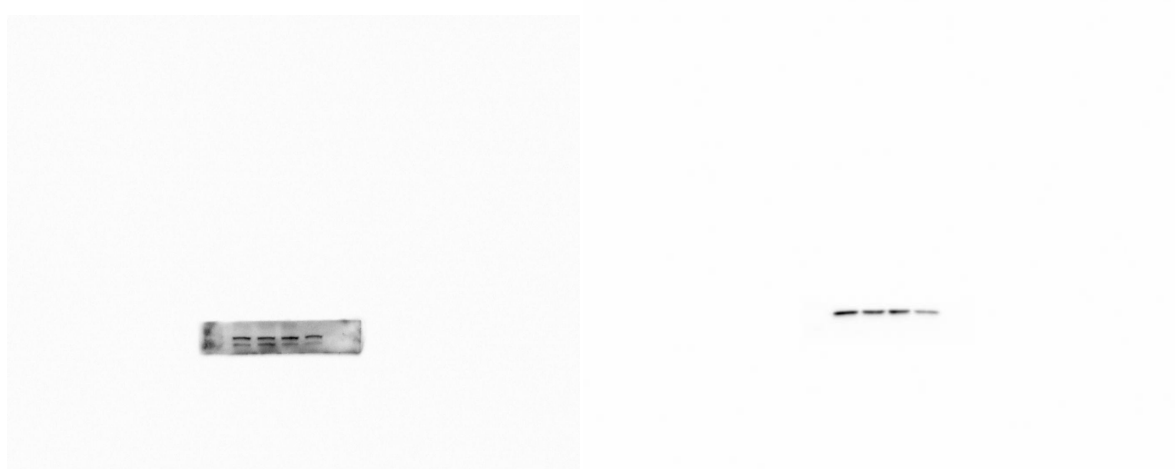

1-1

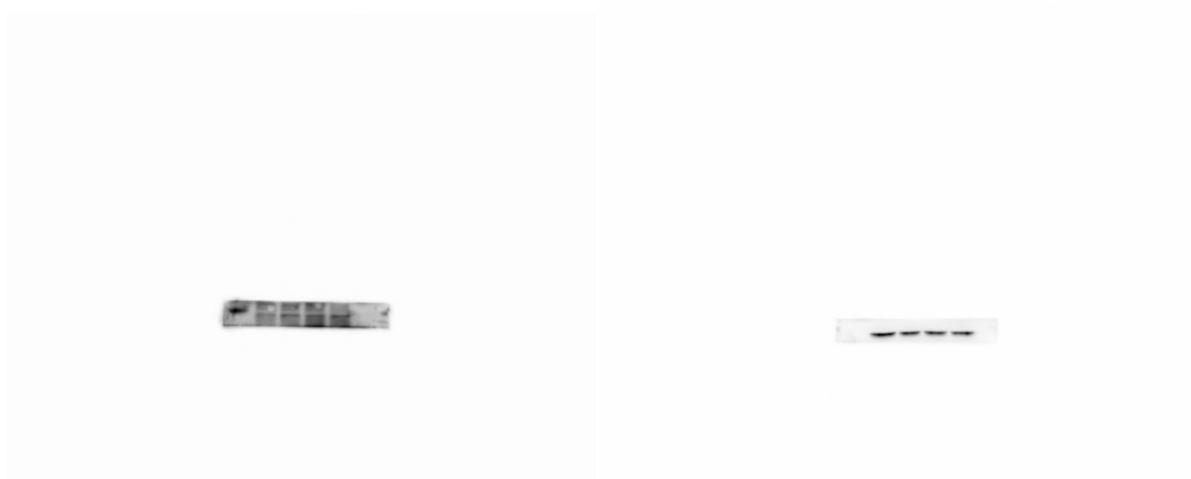

4-2

1000

1000

Supplement: Supplementary file 2 — Western Blots [file 41392_2024_2020_MOESM2_ESM.pdf]
